# A novel mammalian glucokinase exhibiting exclusive inorganic polyphosphate dependence in the cell nucleus

**DOI:** 10.1016/j.bbrep.2017.09.004

**Published:** 2017-09-20

**Authors:** Antasar Ali, D. Claire Wathes, Angelina Swali, Helena Burns, Shamus Burns

**Affiliations:** a Biological Sciences, University of Huddersfield, Huddersfield UK HD1 3DH, United Kingdom; b Department of Pathobiology and Population Sciences, The Royal Veterinary College, London UK NW1 0TU, United Kingdom; c School of Biosciences, University of Nottingham, Nottingham UK LE12 5RD, United Kingdom

**Keywords:** HK, hexokinase, ATP-GK, glucokinase (mammalian ATP utilizing), PPGKm, polyphosphate dependent glucokinase (mammalian), Glucokinase, Hexokinase, Polyphosphate, Evolution

## Abstract

**Background:**

Hexokinase and glucokinase enzymes are ubiquitously expressed and use ATP and ADP as substrates in mammalian systems and a variety of polyphosphate substrates and/or ATP in some eukaryotic and microbial systems. Polyphosphate synthesising or utilizing enzymes are widely expressed in microbial systems but have not been reported in mammalian systems, despite the presence of polyphosphate in mammalian cells. Only two micro-organisms have previously been shown to express an enzyme that uses polyphosphate exclusively.

**Methods:**

A variety of experimental approaches, including NMR and NAD-linked assay systems were used to conduct a biochemical investigation of polyphosphate dependent glucokinase activity in mammalian tissues.

**Results:**

A novel mammalian glucokinase, highly responsive to hexametaphosphate (HMP) but not ATP or ADP as a phosphoryl donor is present in the nuclei of mammalian hepatocytes. The liver enzyme exhibited sigmoidal kinetics with respect to glucose with a S_0.5_ of 12 mM, similar to the known kinetics of mammalian ATP-glucokinase. The Km for HMP (0.5 mM) was also similar to that of phosphoryl donors for mammalian ATP-glucokinases. The new enzyme was inhibited by several nucleotide phosphates.

**Conclusions:**

We report the discovery of a polyphosphate-dependent enzyme system in mammalian cells with kinetics similar to established ATP-dependent glucokinase, also known to have a nuclear location. The kinetics suggest possible regulatory or redox protective roles.

**General significance:**

The role of polyphosphate in mammalian systems has remained an enigma for decades, and the present report describes progress on the significance of this compound in intracellular metabolism in mammals.

## Highlights

• The first mammalian enzyme activity using polyphosphate as a phosphorylation substrate.
• A polyphosphate dependent glucokinase with kinetics similar to human glucokinase.
• Further evidence of discreet substrate specificity of hexokinases.
• A possible evolutionary link between polyphosphate and ATP utilization.

## 1 Introduction

Hexokinases are ubiquitously expressed and have a central role in metabolism phosphorylating hexoses (e.g. glucose), raising the hexose free-energy content and facilitating subsequent reactions central to metabolic energy production and biosynthesis via NADPH production. Hexokinases have undergone extensive evolution resulting in highly specialized enzymes and isoenzymes that have distinct intracellular location, substrate specificity and kinetics [1], [2]. This has resulted in highly specialized functions and accessory actions such as the key components of the glucose sensor in the pancreas and brain [3], [4]. The majority of hexokinases studied use ATP exclusively as the phosphoryl donor, though a novel mammalian hexokinase, expressed in rat and man, with a different evolutionary origin uses ADP [5]. Many bacteria and archaea also synthesise and use a variety of polyphosphate substrates [6], [7] which are considered evolutionary starting points for the ATP-dependent enzymes. Polyphosphate chains may contain 1000 inorganic phosphates joined by high energy phospho-anhydride bonds; they are readily formed under high temperatures, or by dehydration of inorganic phosphate, and would have been an available source of energy before ATP became ubiquitous [8]. Polyphosphate is present at varying concentration in all mammalian cells studied, although no enzymes have been identified to date responsible for either its synthesis or utilization [9]. Two organisms, a phosphate accumulating bacterium, *Microlunatus phosphovorous* [10] and a nitrogen-fixing cyanobacterium, *Anabaena sp.* PCC 1720, express a hexokinase which exclusively uses inorganic polyphosphate and, importantly, cannot phosphorylate hexoses with ATP or ADP [11]. We now report on a mammalian hexokinase which phosphorylates glucose exclusively using inorganic polyphosphate, and which is inhibited by ATP. Inorganic polyphosphate is often concentrated in the hepatocyte nucleus [12], with a variety of roles proposed, such as acting as a chaperone for nuclear proteins, but until now it has not been shown to be of metabolic significance [12]. The enzyme reported here is the first mammalian enzyme shown to utilize polyphosphate in a biochemical reaction. The enzyme is expressed predominantly in the nucleus of hepatocytes, but is also present in cardiac and striated muscle. Because of the enzyme's kinetics we have named it polyphosphate dependent GlucoKinase, mammalian, or PPGKm.

## 2 Methods

### 2.1 Tissue samples

Tissue samples were used immediately after being transported on ice, or from frozen – no significant difference was found in activity or kinetics from Hexokinase (HK), Glucokinase (GK) or polyphosphate dependent glucokinase (PPGKm) using frozen tissue. Only tissues from adult animals were studied: rat, ovine, bovine and porcine liver was investigated for hexokinase activity as well as a variety of ovine tissues including, heart, muscle, lung, kidney, spleen and adipose tissue. Local and National procedures were followed using University Ethics committee approval for the use of animal tissues, and fulfilling the 2010/63/EU directive and UK Home Office regulations.

#### 2.1.1 Tissue homogenates

Tissue homogenates (10% w/v) were prepared in 50 mM HEPES buffer containing 2.5 mM DTE, 7.5 mM MgCl_2_, 100 mM KCl, adjusted to PH 7.4 and kept on ice or frozen at −80 °C. Homogenates were ultra-centrifuged at 100,000 g in a Beckman Coulter ultra-centrifuge model Ti 50,000 at 4 °C, and the supernatant removed and stored on ice or frozen at −80 °C. For direct enzyme assay the supernatant was used without modification. For separation procedures the supernatant was then concentrated using Vivaspin® Centrifugal Concentrators at 4 °C with a 30 kDa MW cut-off, and stored at −80 °C for further purification steps (below).

### 2.2 NAD-linked hexokinase/glucokinase assay

Enzyme activity was determined as previously described exploiting the well established difference in K_m_ and S_0.5_ between hexokinases and glucokinase respectively to obtain their activities. ATP, ADP, CTP, GTP or HMP (sodium hexametaphosphate, 96% Sigma-Aldrich) were used at 5 mM where indicated (all reagents sourced from Sigma-Aldrich). In previous work, the reaction blank for both HK and GK was the reaction system without ATP using 0.5 and 100 mM glucose respectively. Because of the native polyphosphate present in various tissues, the blank for HK was used for the ‘GK’ and PPGKm assays - the rationale being that if the enzyme under consideration is ATP dependent then the concentration of glucose in the system should be irrelevant in the absence of ATP. The fact that increased activity was present in the absence of ATP, but with increasing glucose concentration clearly indicates an additional activity. This activity was lost when G6PDH was removed from the system, on heating and on omission of magnesium. The section on Nuclear Magnetic Resonance (NMR) identifies the mechanism of this reaction. G6PDH from *Leuconostoc mesenteroides* (Sigma-Aldrich) was used which uses NAD as a co-factor. All enzyme activity assays were conducted in triplicate at 37 °C.

### 2.3 Purification steps and procedures

Precast Run Blue tris-glycine 4–12% gradient SDS-PAGE gels and the relevant running and loading buffers were purchased from Expedion Ltd. Spectra™ Multicolour Broad Range Ladders were purchased from Life Technologies. The protein ladder was thawed at room temperature, with 6 µl samples loaded directly into the first lane of the gradient SDS-PAGE gel. All samples were mixed 1:4 with the loading buffer, heated to 100 °C for 10 min and loaded onto the SDS-PAGE gel. The samples were electrophoresed for 1 h at a 120v using an XCell *Surelock*® Mini-Cell from Life Technologies at room temperature.

#### 2.3.1 Chromatography

All enzyme purification steps were conducted with AKTA smart FPLC system (GE Healthcare Life Sciences). Each step was examined for separation suitability and fractions were further analysed for HK and PPGKm activity. All columns were run at 1 ml/min with the exception of the size exclusion column (0.5 ml/min) at room temperature.

##### 2.3.1.1 Anion exchange chromatography

A strong anion exchange media, Q HP, acquired as a pre-packed 1 ml column (GE Health care Life Sciences), was equilibrated with 10 column volumes of buffer A (50 mM HEPES, PH 7.4). Concentrated sheep liver extract (5 ml) was injected onto the column at a rate of 1 ml/min. An initial wash step with homogenization buffer was carried out for a further 5 ml, followed by a gradient elution of protein fractions using the same buffer over 30 min.

##### 2.3.1.2 Size exclusion chromatography

HiPrep 16/60 Sephacryl S-200 h pre-packed gel filtration columns with a bed volume of 120 ml were used. The columns were equilibrated with two column volumes of homogenization buffer. A spin concentrated 500 µl sample was injected into the column and automatic peak elution was set at 30 mAU running for 8 h at 4 °C.

##### 2.3.1.3 Hydrophobic interaction chromatography

HiTrap Butyl FF prepacked Butyl Sepharose 4 Fast Flow columns were equilibrated with 10 column volumes of buffer (3 M NaCl, 50 mM HEPES, PH 7.4). The test samples were buffer exchanged with this buffer before loading the column. An initial wash step with the same buffer was followed by gradient elution of protein fractions with NaCl-free buffer (50 mM HEPES, PH 7.4) over 30 min.

### 2.4 Substrate depletion on purified fractions

Supernatant was polyphosphate substrate depleted by incubation with 100 mM glucose, 5 mM NAD and 8 units G6PDH in homogenization buffer until activity tended to zero (approx.12 h at 37 °C). Addition of 2 mM HMP restored activity.

### 2.5 Cell fractionation

Three buffers all adjusted to pH 7.4 were used in the cell fractionation procedures as follows: **Buffer 1** contained 0.25 M sucrose, 5 mM Tris, 7.5 mM MgCl_2_ and 1 mM Dithiothreitol (DTT). **Buffer 2** was identical to Buffer 1 but contained 2 M sucrose instead of 0.25 M sucrose. **Buffer 3** contained 7.5 mM MgCl_2_, 2.5 mM DTT, 100 mM KCl and 50 mM Na HEPES. Liver (10 g) was homogenized at 4 °C in 50 ml Buffer 1 using a pestle and mortar for 10 min. The suspension was filtered through muslin cloth and the homogenate spun in a 50 ml centrifuge tube at 600 g for 10 min. The supernatant was retained (cytoplasmic fraction) and the pellet re-suspended (nuclear fraction) in 6 ml of Buffer 2. The nuclear fraction was centrifuged at 800 g (Beckman coulter Avanti J-26XPI centrifuge S/N JXT08C02) for 15 min. The supernatant was discarded and the nuclear pellet re-suspended in Buffer I, and kept on ice (nuclear fraction). The cytoplasmic fraction was spun at 8000 g (using Beckman coulter Avanti J-26XPI centrifuge S/N JXT08C02) for 10 min: the supernatant was saved and the pellet re-suspended (mitochondrial fraction) in 3 ml of homogenization Buffer I. The remaining supernatant was then centrifuged at 15,000 g (Beckman coulter Avanti J-26XPI centrifuge S/N JXT08C02) for 10 min, The pellet (lysosomal fraction) was re-suspended in 6 ml of homogenization buffer 3, the final supernatant and the nuclear pellet were centrifuged at 100,000 g for 1 h (using Beckman coulter optima L-look ultra-centrifuge 52, Ti rotor S/N colo8E23). After the last spin, 3 × 1 ml aliquots of particle-free supernatant were collected carefully avoiding the upper fatty layer and stored at −80 °C and the pellet was re-suspended (microsomal fraction) in 3 ml of homogenization Buffer 3. The nuclear pellet was re-suspended in 6 ml of Buffer 3.

The nuclear and the mitochondrial fractions were sonicated on ice for a total of 20 s, at the highest speed with 5 s intervals to rupture the nuclear and mitochondrial membranes and release the soluble proteins. Then, both fractions were centrifuged at 4 °C for 30 min at 9000 g (Beckman coulter Avanti J-26XPI centrifuge S/N JXT08C02) to remove membrane debris. The supernatants were collected and the pellets discarded. All aliquots were examined for PPGKm and hexokinase activity as described above. All subcellular fractions were assayed for an appropriate marker enzyme to ascertain their purity as previously described [13] as follows: *mitochondria,* glutamate dehydrogenase; *lysosomes*, acid phosphatase; *microsomes*, glucose 6-phosphatase; *particle free supernatant*, lactate dehydrogenase. Further details are available on request to the corresponding author.

### 2.6 Protein identification by LC-MS/MS

In-gel digestion with trypsin was performed after reduction with DTE and S-carbamidomethylation with iodoacetamide. Gel pieces were washed two times with 50% (v:v) aqueous acetonitrile containing aqueous 25 mM ammonium bicarbonate, then once with acetonitrile before drying in a vacuum concentrator for 20 min. An 0.2 µg aliquot of sequencing grade modified porcine trypsin (Promega) was added in 10 µl aqueous 25 mM ammonium bicarbonate prior to incubation overnight at 37 °C.

Analysis was performed by LC-MS/MS following in-gel digestion with trypsin. Peptides were eluted over a 20 min gradient using a Waters nanoAcquity UPLC interfaced to a Bruker maXis HD mass spectrometer. Peptide identification was achieved by searching tandem mass spectra against the expected protein database using the Mascot search program described below. Matches were filtered to accept only peptides with expect scores of 0.005 or better. Protein inferences required a minimum of two unique peptide sequences.

Tandem mass spectra were searched against the *Ovis* subset of the NCBI database (147,277 sequences; 77,737,218 residues) using a locally-running copy of the Mascot program (Matrix Science Ltd., version 2.5.1), through the Bruker ProteinScape interface (version 2.1). Search criteria specified: Enzyme, trypsin; Fixed modifications, Carbamidomethyl (C); Variable modifications, Oxidation (M), Deamidation (N,Q); Peptide tolerance, 10 ppm; MS/MS tolerance, 0.1 Da; Instrument, ESI-QUAD-TOF.

### 2.7 Glucokinase NMR assays to establish the mechanisms of glucose phosphorylation and its relationship to ATP-dependent glucokinase

NMR samples were run on a Bruker Avance 500 NMR Spectrometer using a 5 mm Broadband BBO probe with z-gradients and ATMA (autotune and match). NMR 5 mm tubes were loaded with 600 µl of homogenate and 20 iu of G6PDH, 99% ^13^C_1_-glucose, 2 mM HMP or 5 mM ATP in homogenizing buffer were added as required. Experiments were started with the addition of glucose or G6PDH depending on the experimental requirements. The increased volume of homogenate, and hence enzyme was necessary to account for the lower sensitivity of the ^31^P NMR assay. The reactions were monitored over 4 h. ^13^C and ^31^P NMR spectra were recorded in an interleaved fashion such that blocks of data could be merged with comparable time stamps for comparison. Spectra were recorded fully relaxed using 30° pulses and a 5 s recycling time to satisfy quantitative requirements. ^13^C and ^31^P spectra were acquired proton decoupled to maximise sensitivity.

## 3 Results and discussion

### 3.1 Polyphosphate dependent glucose 6-phosphorylating activity in liver

**Fig. 1:**
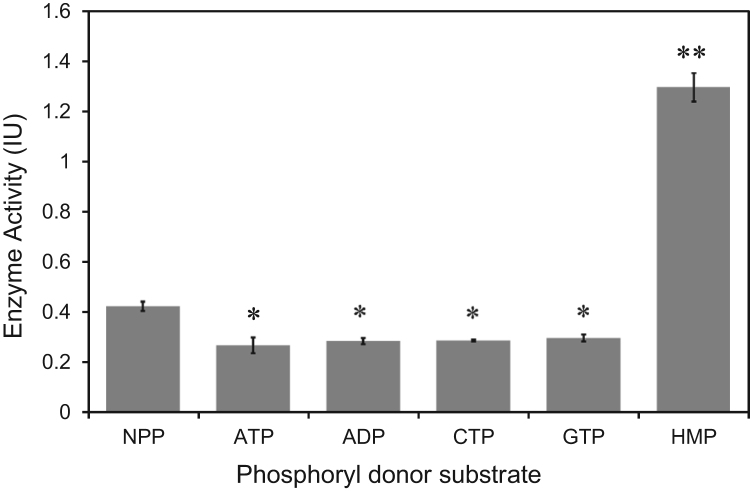
Glucose 6-phosphorylating activity of a novel polyphosphate dependent glucokinase in mammalian liver using different phosphorylation substrates. Results are mean ± sem (n = 3 replicates) with * indicating p < 0.05, and **p < 0.01. Data shown are from ovine liver. In the absence of ATP the enzyme was capable of utilizing an inherent bound polyphosphate (NPP) substrate to covert glucose to glucose 6-phosphate. ATP and other nucleoside phosphates (5 mM) inhibited the enzyme, whilst 5 mM inorganic polyphosphate as hexametaphosphate (HMP) resulted in markedly increased activity. Significant differences indicated to NPP. 1 international enzyme unit (IU) = 1.0 µmol G6P formed per min per g liver.

Whilst investigating the adaptions of liver function during pregnancy we observed a very high activity of hexokinase in bovine and ovine tissue extracts which was ATP-independent and only present at high concentrations of glucose (above 10 mM). Following the discovery of a mammalian ADP-dependent hexokinase [5] we decided to screen tissues for enzyme activity with ADP and a variety of phosphorylation substrates, using a standard glucokinase (GK) assay protocol [14], [15] but accounting for the ATP-independent activity which had previously been mistakenly used as a blank in the GK assay [16]. Since inherent activity of the enzyme might arise from native polyphosphates already in the liver [17], we included inorganic polyphosphates in this screen. The liver enzyme was shown to be highly responsive to inorganic polyphosphate, particularly hexametaphosphate (Fig. 1).

The enzyme showed significant inhibition by ATP and other nucleotides (Fig. 1) Addition of 5 mM ATP, ADP or AMP had similar effects on lowering activity (P < 0.01) suggesting the nucleoside moiety itself was responsible for lowering activity. Qualitatively similar data were obtained from other species (Fig. 3C) with the highest activity in ovine liver and lowest in rat liver. The majority of known polyphosphate dependent hexokinases are also capable of using ATP as a substrate. However, only two organisms have been reported to express hexokinase enzymes that use polyphosphate exclusively as a substrate [10], [11]. The structural basis of restricting the active site to polyphosphate, and preventing ATP utilization is currently unknown, and significant homology between the exclusive and inclusive polyphosphate and ATP utilizing enzymes raises the question of how this is achieved. Mammalian hexokinase isozymes 1–3 (HK1-3) are allosterically inhibited by the product, glucose 6-phosphate [2]. Nucleoside phosphate sensitive allosteric sites are common on metabolically regulated enzymes conferring, for example, cellular energy charge [2], but the precise role of nucleoside sensitivity in the new enzyme merits further study.

### 3.2 Kinetics of the ATP independent polyphosphate dependent glucokinase

**Fig. 2:**
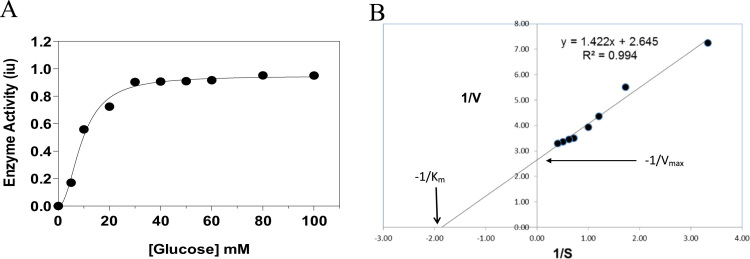
Enzyme kinetics with respect to (A) glucose and (B) polyphosphate Results are mean ± sem (n = 3 replicates). PPGKm displayed sigmoidal kinetics with respect to glucose (A), with a high S_0.5_ of 12 mM, making it a true glucokinase potentially sensitive to physiological glucose concentrations. The Vmax of the enzyme and its phosphorylating capacity are in the same order as human and rat glucokinase in liver. Sigmoidal fit using SigmaPlot's enzyme kinetics module. (B) An inverse plot of 1/v (enzyme velocity versus 1/s (substrate - hexametaphosphate concentration with glucose constant at 10 mM). Classical Michaelis-Menten kinetics for HMP gave a Km of 0.5 mM, comparable to the Km for ATP of that of mammalian ATP-GK.

Mammalian ATP-dependent glucokinase does not follow Michaelis-Menten kinetics, but shows a sigmoidal response to glucose. K_m_, the half-saturating substrate concentration which indicates substrate affinity for a Michaelis-Menten enzyme is replaced with the analogous S_0.5_ for a sigmoidal enzyme. There was a sigmoidal response to increasing glucose concentration and high half-saturating glucose kinetics (S_0.5_ for glucose = 12 mM) (Fig. 2A) for the polyphosphate dependent glucokinase. The low affinity for glucose is similar to mammalian ATP-dependent glucokinase (hexokinase type 4, commonly referred to as glucokinase or GK) which also shows a sigmoidal response to glucose as a monomeric enzyme with an S_0.5_ of 8 mM. These two features, inhibition and sigmoidal kinetics with respect to glucose might indicate strong similarity to mammalian glucokinase [18], [19], [20]. Polyphosphate entry to the active site has been proposed to arise via a sequential mechanism (11) but evidence is presented in this study that polyphosphate is already tightly bound to the enzyme (see 3.4. below). The kinetics with respect to polyphosphate (Fig. 2B) suggest that the enzyme may be responsive to local, possibly bound polyphosphate, as the K_m_ is similar to that of mammalian hexokinases K_m_ for ATP and higher (500 μM) than that reported in microbial systems (2 μM – Ref. [11]).

### 3.3 Subcellular, tissue and species distribution of PPGKm

**Fig. 3:**
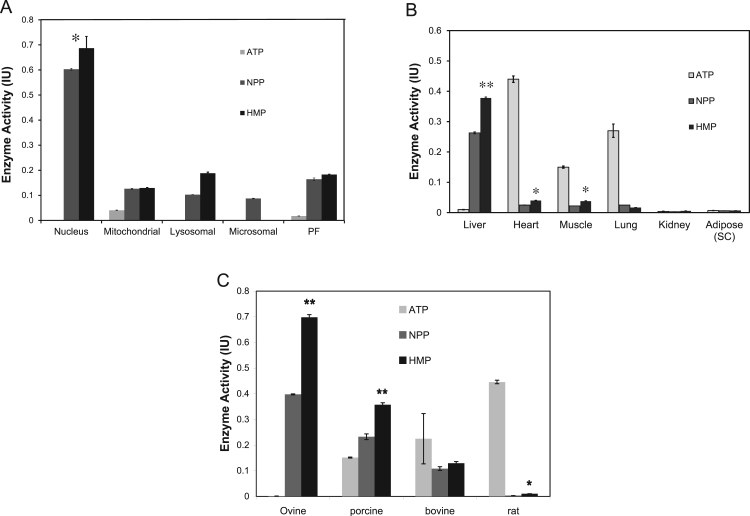
The polyphosphate dependent glucokianse retained its native substrate in the nucleus (A) of the hepatocyte, but remained highly responsive to exogenous HMP. PF = Particle-Free supernatant. Tissue (A) and (B) species distribution suggest widespread expression consistent with long standing observations of polyphosphate presence in mammalian cells. In the tissue and species (C) studied PPGKm remained responsive to external HMP indicated by asterisks, but also showed some native activity (NPP).

The inhibition of PPGKm by ATP suggested an alternative biochemical mechanism to GK, which involves an inhibitory regulatory protein (GKRP), so we next investigated the subcellular distribution of enzyme activity in liver. Mammalian GK resides in the hepatocyte nucleus during fasting and its activation and release from an inhibitory binding protein leads to cytosolic translocation and phosphorylation of glucose in the cytoplasm [20]. The high S_0.5_ and low glucose affinity of ATP-GK ensures that enzyme activity increases in line with glycemia and metabolism of glucose during feeding. These kinetic properties give the enzyme key roles in the brain, liver and pancreas [20]. The low affinity for glucose of the novel polyphosphate dependent enzyme described here would confer a similar capacity on PPGKm and its total cellular activity is of the same order. Cell fractionation studies showed that the enzyme was highly concentrated in the hepatocyte nucleus (Fig. 3A). During fasting ATP-GK is known to accumulate in the hepatocyte nucleus, but does not normally phosphorylate glucose there [21]. Activity of ATP-GK is not found outside of the liver, pancreatic ß-cell and brain: in contrast PPGKm was present in striated muscle and was also found at a significant concentration in cardiac tissue and lung (Fig. 3B). Activity was very low in adipose tissue and kidney, and unmeasurable in spleen. A preliminary investigation into the species wide distribution of PPGKm activity indicated that it was at high activity in ovine, bovine and porcine liver (Fig. 3C). Activity was also present in sufficient quantities in rat liver to warrant a re-appraisal of hexokinase measurements in this species, since the presence of polyphosphate-dependent high K_m_ (S_0.5_) enzymes would interfere with existing HK/GK measurement techniques and strategies, particularly in the liver.

### 3.4 Partial purification suggests molecular characteristics distinct from existing hexokinases

**Fig. 4:**
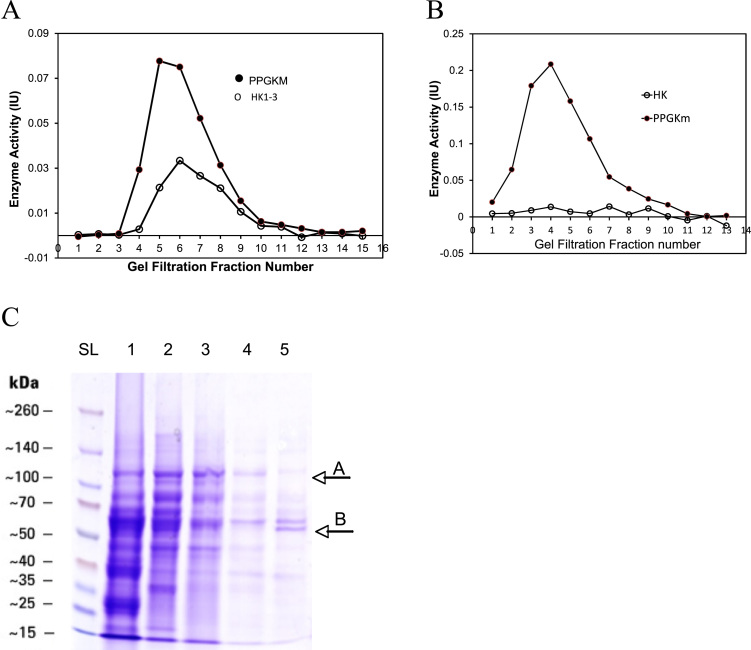
Molecular characteristics and partial purification of PPGKm from liver. (A) Gel filtration indicated that PPGKm has a higher molecular weight than Hexokinases 1–3, consistently eluting before hexokinase. (B) The nuclear fraction showed only polyphosphate dependent activity, with no ATP-dependent activity detectable reinforcing data in Fig. 1. (C) SDS-PAGE gel of a 5-stage purification process: *lane-1*, ultra-centrifuged intact ovine liver homogenate; *lane-2*, the PPGKm active fraction eluted from anionic exchange chromatography, Fraction ‘P2’. This fraction was bound very strongly to a positively charged column and would not bind to a negatively charged one, indicating that the active enzyme protein carried a high negative charge, likely to be bound polyphosphate; *lane-3*, the active fraction following ‘P2’ addition to a gel filtration (size exclusion) column – now called fraction ‘P3’; *lane-4*, hydrophobic column chromatography of active fraction ‘P3’ – the active enzyme fraction showed very weak interaction with the column consistent with a highly hydrophilic, charged protein. Two distinct bands were visible at 110 and 55 kDa, suggesting a dimer. *Lane-5*, the fraction used for Lane-4 was substrate depleted by running against saturating glucose without exogenous polyphosphate for 12 h after which time activity plateaued to zero. Two bands remained visible, but the large Mw band was replaced by a 55 kDa band (A ⇒ B). These results might suggest a heterodimer or that bound polyphosphate alters the observed Mw. Activity in this fraction could be readily restored via the addition of exogenous HMP, so the gel shift was not the result of non-functioning enzyme.

Gel filtration (Fig. 4(A) and (B)) indicated that PPGKm has a higher molecular weight (Mw) than hexokinases I-III with ‘fraction 3 and 4’ displaying a high activity of PPGKm but no low Km hexokinase activity. PPGKm exhibited a larger range of molecular weights (fractions 4–12) than the low Km hexokinases (fractions 5–11). Gel filtration of the nuclear fraction obtained from cell fractionation (Fig. 4A) reinforced this conclusion and also indicated a broader molecular weight profile, with no low K_m_ hexokinases detected. We postulated that a varied chain length of the polyphosphate substrate tightly bound to the PPGKm might account for this Mw variation. To investigate this, further separation procedures were conducted using anionic exchange, hydrophobic column chromatography and SDS-PAGE (Fig. 3C). As activity was retained at every stage of separation we decided to substrate deplete the partially purified enzyme of polyphosphate and re-analyze with PAGE (Fig. 3C). The substrate depleted enzyme showed no activity in the presence of 100 mM glucose, but activity was readily restored by the addition of HMP (data not shown).

During the process of partial purification two principle bands were visible in the fraction with enzyme activity (Fig. 4C lane 4) at approximately 55 kDa and 110 kDa (arrows A and B), possibly suggesting that the enzyme can dimerize like HKs, but can also function as a monomer following substrate depletion (lane 5). These results may help explain the molecular weight range observed in gel filtration compared with HK1-3 (Fig. 2A and B). The appearance of a new lower molecular weight band following substrate depletion (lane 5, arrow B) may be the result of the tightly bound polyphosphate being lost during the process of substrate exhaustion, resulting in an enzyme with an apparent molecular weight lowered by a few kDa. Each kDa of molecular weight is equivalent to 10 orthophosphate moieties lost from the enzyme during substrate depletion. An alternative interpretation is that the structure of the protein is altered during substrate depletion resulting in a structure or amino acid residue driven gel shift. Unexplained gel shifts of proteins with known molecular weights and structures has highlighted the role of specific domains and residues [22] as well as post-translational modification significantly influencing the extent of SDS-binding and causing characteristic, predictable SDS-PAGE gel shits [23]. Tight polyphosphate binding could result in gel shifts if it is retained during SDS treatment in this way. Following substrate depletion, PPGKm activity could be readily restored by the addition of 2 mM HMP to the assay system, indicating that the enzyme was functioning normally but that the bound polyphosphate had been exhausted, or chain-shortened such that it was inaccessible to the active site. The progressive application of chromatographic techniques revealed several biochemical and biophysical properties of PPGKm. Elution on an anionic column indicated that the enzyme was highly negatively charged, possibly as a result of tight polyphosphate binding. The enzyme showed very little interaction with a hydrophobic column suggesting it has no major exposed hydrophobic domains. Gel filtration indicated that the polyphosphate dependent glucokinase was larger than HK1-3, with bands in the SDS-PAGE gel suggesting a dimer with a Mw of 110 kDa. Substrate depletion indicated that polyphosphate may account for a significant proportion of the molecular weight and that it may not bind equally to both polypeptide chains.

### 3.5 NMR based enzyme assays define the glucose 6-phosphorylation mechanism of PPGKm

**Fig. 5:**
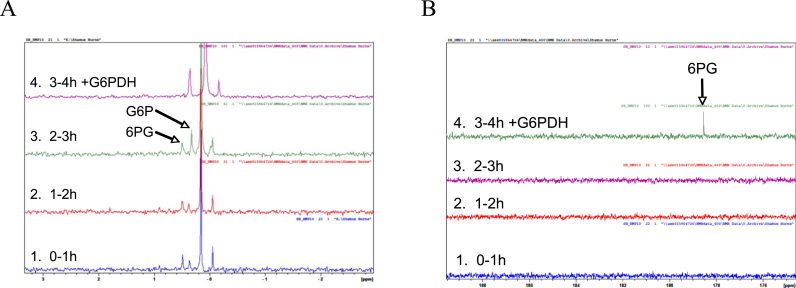
(A) ^31^P NMR and (B) ^13^C NMR spectra acquired during the HMP assay of PPGKm in the presence and absence of the linking enzyme G6PDH and 100 mM glucose labelled 99% ^13^C at carbon 1. G6P formation occurred slowly in the absence of G6PDH. In the absence of G6PDH 6-phosphogluconate was below detection with ^13^C NMR, but, on addition of G6PDH rapid oxidation of the C1 of glucose 6-phosphate occurred, producing the carboxylic acid group of 6-phosphogluconate at C1. Similar results were obtained using recombinant glucokinase. 6PG was only formed at 100 mM glucose and not at 0.5 mM glucose. These data confirm that the enzyme mechanism involves the same intermediates and follows the same biochemical mechanism for ATP-GK and PPGKm except that the latter only uses PolyPi and not ATP to achieve glucose 6-phosphorylation in the presence of 100 mM glucose.

Using ^31^P NMR it was possible to show that PPGKm metabolized HMP in a similar way to its ATP dependent analogues GK and HK metabolizing ATP (Fig. 5). In the absence of G6PDH, the linking enzyme in the NADH assay, little G6P was formed even after 3 h (Fig. 5). This occurred at a similar rate to HK but slower than GK, suggesting negative feedback by G6P, a classical feature of the dimeric low Km HKs [2]. On addition of G6PDH large amounts of 6-phosphogluconate (6PG) were formed as expected in both ATP and HMP driven reactions. In the HMP assay, ATP was absent so the formation of phosphorylated glucose moieties (G6P and 6-phosphogluconate – 6PG) must have arisen from HMP derived orthophosphate moieties. This was reinforced in the ^13^C spectra (Fig. 5b): the glucose used in the experiment was labelled with 99% ^13^C at C_1,_ so the appearance of the peak at 178.5 ppm confirms that the aldehyde group C_1_ had been oxidised to a carboxylic acid with a concomitant chemical shift in the NMR spectrum from 97 and 99 ppm (alpha and beta anomers at C_1_ in glucose) – i.e. the conversion of glucose to gluconate. The chemical shift of the 6PG peak was confirmed in the samples by spiking them with known standards, and PPGKm produced identical results to hepatic ATP dependent GK, when 100 mM glucose and 5 mM ATP were used in the absence of HMP. These results reinforced the conclusion that the enzyme mechanism conducted by PPGKm and ATP-GK are similar, both producing G6P from glucose, but that PPGKm is a polyphosphate dependent glucose 6-phosphotransferase.

The partial purification procedure shown in Fig. 4C was used to obtain LC-MS/MS fingerprinting on two occasions at two separate stages. The bands in Lane 5 indicated homology only to catalase. Following gel filtration and SDS-PAGE of the nuclear fraction derived from the cell fractionation studies, the band at 110 kDa was fingerprinted with MS/MS. This indicated the presence of a number of proteins but no established HK or GK protein homology was identified. HK homology in this band was to that of HSP70 (A1/B1) which has an ATP/ADP binding site and a nuclear localization. HSP70 is known to bind polyphosphate [12] but recombinant HSP70 did not show PPGK activity in the presence or absence of HMP (including after overnight incubation with 2 mM HMP – Ali et al. unpublished data). Whether post-translational modification or complexing of HSP70 results in the activity described is currently unknown.

### 3.6 Substrate specificity of the hexokinase active site

It is likely that polyphosphate was an early phosphorylation substrate during the emergence of life before ATP synthesis became widespread [8]. It was present in all the mammalian cells studied and has been shown to be critical in a number of cellular processes, such as the activation of mTOR, demonstrated in mammary cancer cells [24]. Identification of the PPGKm gene is a critical next step in understanding the role of this enzyme in mammalian cells. One fundamental question is how the active sites of hexokinases are organised to accept either ATP and/or polyphosphates, especially those that use ATP alone or polyphosphate alone. An understanding of how ATP can be specifically excluded as a substrate may help in understanding hexokinase evolution and early phospho-anhydride biochemistry. Inhibition of hexokinases is of interest in cancer research and the specificity of inhibitors with respect to ATP, ADP or polyphosphates amongst the hexokinase family may help to inform inhibitor design and emerging strategies in cancer hexokinase inhibitor therapeutics [25].

## 4 Conclusions

A third example of a hexokinase using polyphosphate exclusively as a phosphoryl donor has been identified, this time in mammalian tissues. This is the first polyphosphate utilizing enzyme activity demonstrated in mammalian cells as far as we are aware. The evolutionary significance of this enzyme may help in the understanding of the shift of substrate specificity of hexokinases from polyphosphate to ATP. The enzyme kinetics are similar to glucokinase as defined by its high S_0.5_ for glucose. Preliminary analysis suggests that the enzyme tightly binds polyphosphate which can act as a native substrate for glucose 6-phosphorylation. Because of the finite supply of polyphosphate in cells, the enzyme system is likely to be involved in acute cellular events. An obvious possibility, though speculative, is the supply of increased reductive capacity in the form of NADPH for glutathione synthesis during oxidative stress, supported by the observation that G6PDH localizes to the nuclear periphery [26].

## Author contribution

Antasar Ali, Angelina Swali and Helena Burns performed the experiments. Shamus Burns made the initial observations of the enzyme activity and designed the experimental approach. Claire Wathes provided funding and support for animal tissues.

## Author information

The authors declare no competing interests. Information requests, reprints and questions should be addressed to the corresponding author, S.B. (s.p.burns@hud.ac.uk).

## Notes

A naming conflict for the glucokinases presently exists because mammalian biologists have named HK4, ‘glucokinase’, by virtue of its pivotal role in insulin secretion, hepatic glucose handling and brain glucose sensing: in short, glycemic regulation. Microbiologists apply the name ‘glucokinase’ to enzymes that show a high specificity to glucose compared with other sugars, an important characteristic of microbial hexokinases. The term ‘glucokinase’ for the novel enzyme here follows the mammalian naming convention with reference to its kinetics for glucose (low affinity), rather than its substrate specificity.
